# Evidence of West Nile virus exposure in healthy donors and a clinical case in an immunocompromised child: emerging public health implications

**DOI:** 10.3389/fmed.2026.1744989

**Published:** 2026-04-01

**Authors:** Ashwaq M. Al-Nazawi, Rana Alghamdi, Ali A. Al-Zahrani, Wafa Ali Hetany, Maseer Khan

**Affiliations:** 1 Epidemiology Program, Department of Public Health, College of Nursing and Health Sciences, Jazan University, Jazan, Saudi Arabia; 2 Laboratory Department, Jazan University Hospital, Jazan University, Jazan, Saudi Arabia; 3 Vector-Borne and Zoonotic Diseases Administration, Public Health, Second Health Cluster, Jeddah, Saudi Arabia; 4 General Medical Committee, MOH, Jeddah, Saudi Arabia; 5 Department of Public Health, College of Nursing and Health Sciences, Jazan University, Jazan, Saudi Arabia

**Keywords:** blood transfusion, encephalitis, Ewing’s sarcoma, immunocompromised, Saudi Arabia, West Nile virus

## Abstract

**Background:**

West Nile virus (WNV), an emerging mosquito-borne flavivirus, is a growing global concern, particularly in tropical regions, where climatic conditions favor vector proliferation. Although often asymptomatic, less than 1% of cases develop severe neuroinvasive disease, primarily among the elderly or immunocompromised. Transfusion-transmitted WNV infections have been documented but remain underrecognized in the Middle East.

**Methods:**

This study reports a case of a 4-year-old Saudi girl with Ewing’s sarcoma undergoing chemotherapy who developed WNV encephalitis following multiple blood transfusions from 30 healthy blood donors. The investigation was conducted at a tertiary hospital in Jeddah. Serological screening of 30 blood donors was performed to identify potential sources of infection.

**Results:**

The patient presented with febrile seizures, and MRI findings of bilateral thalamic and midbrain signal changes were consistent with viral encephalitis. WNV infection in the patient was confirmed by the polymerase chain reaction. The clinical picture correlated with reported cases of transfusion-associated WNV infection in immunocompromised hosts. Four of the 30 donor samples (13.3%) tested positive for anti-WNV antibodies, indicating prior exposure among donors and supporting transfusion as a plausible but unconfirmed transmission route, as retrospective molecular testing of implicated blood units was not available.

**Conclusion:**

This case underscores the need to consider the incorporation of nucleic acid testing (NAT) for WNV RNA into pretransfusion screening protocols, particularly for high-risk recipients and during peak transmission periods, to reduce the risk of transfusion-transmitted infection. The detection of anti-WNV antibodies among healthy donors highlights the silent viral circulation in the community. Strengthening national surveillance systems, enhancing vector control measures, and improving clinician awareness are vital to mitigate transfusion-related WNV transmission in endemic and emerging regions.

## Introduction

Several mosquito-borne pathogens, including dengue virus (DENV), chikungunya virus (CHIKV), Japanese encephalitis virus (JEV), Rift Valley fever virus (RVFV), and West Nile virus (WNV), continue to pose significant public health challenges globally. While dengue and chikungunya remain endemic in many regions, the emergence and geographic expansion of WNV and RVFV have further complicated vector control strategies and surveillance systems. Mosquito-borne diseases involve multifaceted ecosystem interactions depending on environmental conditions prevailing in the area (1). West Nile fever is currently the most widespread mosquito-borne fever (2). Climate change is creating conditions that allow mosquito vectors to expand their range, affecting millions of people worldwide (3). West Nile fever is asymptomatic in approximately 75% of cases; however, in 1% of cases, it can cause serious illnesses, such as encephalitis and meningitis, mostly in elderly and immunocompromised individuals, often causing death (4, 5). No vaccine or antiviral treatment is available for West Nile fever, and its increasing occurrence poses a serious threat to people living in tropical climates (4). In Saudi Arabia, evidence of WNV circulation has been increasingly recognized over the past decade. Serological studies have demonstrated WNV antibodies among humans and animals, including horses and pigeons, suggesting silent endemic circulation. A confirmed human case of West Nile encephalitis was reported in Jeddah in 2020 through the Field Epidemiology Training Program investigation, establishing documented neuroinvasive disease within the country.

WNV is an arthropod-borne virus belonging to the Flaviviridae family, and it is a single-stranded RNA virus (6). Infection spreads from wild birds to humans and horses, which are both dead-end hosts, as they typically develop insufficient viremia to sustain further mosquito-mediated transmission (7). Transmission can also occur through blood transfusion, breastfeeding, and needle pricks in hospitals and laboratories (8). Parenteral transmission of WNV is not uncommon, as many such cases have been reported in the past (9). A confirmed case of WNV encephalitis was reported in Jeddah in March 2020 (10). The patient was a known case of primary intracranial sarcoma and had been receiving chemotherapy for 38 months. During the blood transfusions, the patient developed WNV encephalitis and showed symptoms such as seizures, progressing to a semi-comatose state. On investigating the etiology, it was found that WNV was transmitted through blood donation (10). Serological evidence of WNV exposure has been reported in Saudi Arabia, demonstrating detectable anti-WNV antibodies among humans and equine populations. WNV transmission is closely associated with mosquito density and seasonal climatic factors, so screening strategies for blood products should be guided by regional epidemiology and seasonal risk assessment. In areas with documented human or animal seropositivity, particularly during periods of peak mosquito activity, targeted or seasonal nucleic acid testing (NAT) may represent a rational and cost-effective approach (10). Several studies have incidentally identified WNV in blood donations from otherwise healthy individuals (11–13).

Blood transfusion is required for immunocompromised patients. Here, we present a case study of a 4-year-old girl with Ewing’s sarcoma on chemotherapy. The child was given multiple blood transfusions, which might have caused West Nile fever.

## Methodology

This study was conducted at a hospital in Jeddah, where a case of Ewing’s sarcoma was longitudinally followed over a defined period. During management, the patient acquired a WNV infection. Subsequent investigation into the source of infection included serological screening of blood donors, which revealed that 4 of 30 samples were positive for anti-WNV antibodies. All 30 implicated blood donors were retrospectively contacted and screened for WNV infection. Serum samples were collected and tested for anti-WNV IgM and IgG antibodies using a commercially available enzyme-linked immunosorbent assay (ELISA) kit. At the time of transfusion, routine blood bank screening did not include WNV nucleic acid testing (NAT) or serology, as WNV screening is not part of the standard donor screening protocol in Saudi Arabia. Donors were asymptomatic at the time of donation and met standard donor eligibility criteria. Details of plaque reduction neutralization testing (PRNT) were not available from the records.

### Case presentation

A 4-year-old Saudi girl with a history of sinusitis was diagnosed with Ewing’s sarcoma and primitive neuroectodermal tumor (PNET) of the right temporal lobe, which was non-metastatic with negative bone marrow involvement. The patient was a resident of Jeddah, Saudi Arabia, and had no history of recent domestic or international travel in the 3 months preceding symptom onset. According to family reports, she had no known exposure to rural environments, livestock, or equine settings. The patient resided primarily in an urban setting and had limited outdoor activity due to ongoing chemotherapy. No household members reported similar febrile or neurological illness during the same period. Detailed documentation of the specific chemotherapy agents and dosing schedule was not available in the retrieved records for this retrospective analysis.

### Initial surgical management

The patient underwent a right frontoparietal craniotomy with debulking on 04/08/2019 at Khamis Mushait Military Hospital. During hospitalization, the patient was treated empirically for suspected infection; however, detailed microbiological documentation and antimicrobial treatment records were not available for review in this retrospective analysis. Despite surgery, a residual tumor mass remained. A Port-a-Cath was inserted on 04 September 2019 to facilitate chemotherapy.

### Neurological manifestations

During follow-up, the patient experienced recurrent right-sided facial nerve (cranial nerve VII) palsy, presenting as eyelid drooping and corner-of-mouth weakness, with no history of trauma, viral illness, or skin disease. These episodes occurred for 7 months, 4 months, and 1 month prior to 02 January 2020, each treated with prednisolone.

### Mass progression

On 29 January 2020, imaging revealed a right temporoparietal subgaleal mass without evidence of hematoma, abscess, or abnormal enhancement, suggesting tumor regrowth.

### Seizure episodes

The patient experienced her first febrile tonic seizure on 02 February 2020, which she aborted with lorazepam. A second seizure occurred on 04 February 2020 during the administration of Keppra infusion.

### Imaging and viral investigation

MRI of the brain on 05 February 2020 showed bilateral thalamic and midbrain signal changes, suggesting a viral etiology, with flavivirus infection considered clinically. Dengue serology was performed on 06 February 2020 and returned negative on 11 February 2020. WNV serology was sent on 10 February 2020, with polymerase chain reaction (PCR) confirming WNV infection on 22 February 2020. Dengue virus infection was concurrently evaluated using serological testing and yielded negative results, supporting the exclusion of dengue as an alternative flaviviral etiology.

### Investigation of potential source

To investigate the source of WNV infection, serological screening of 30 blood donors was conducted, revealing four positive donors for anti-WNV antibodies.

**Figure 1:**
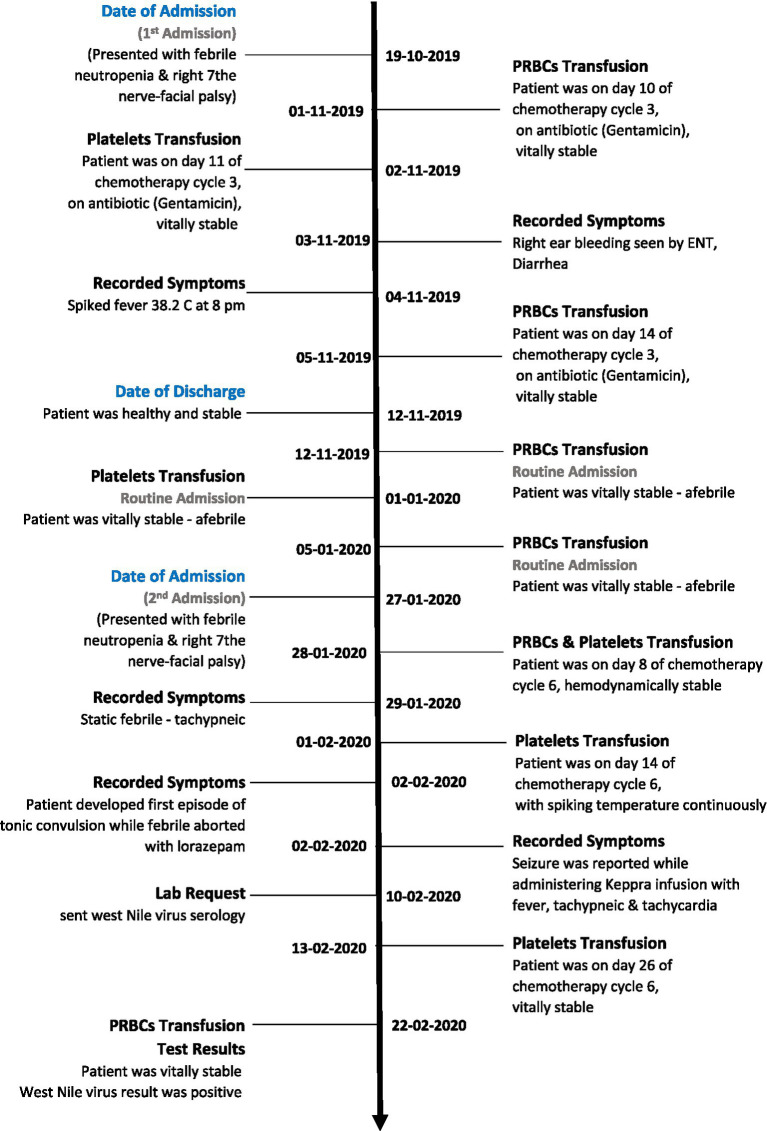
Timeline of blood transfusion with abnormal symptoms or changes recorded.

Diagnostic evaluation included testing for both dengue virus and WNV using serological and molecular assays performed at the treating institution’s reference laboratory. Dengue testing was conducted as part of the initial evaluation for febrile illness and returned negative results. Subsequent testing for WNV demonstrated evidence of infection based on available serological and molecular findings documented in the medical record (Table 1, Figure 1).

**Table 1:** Blood transfusion history with abnormal symptoms or changes after transfusion.

S. No	Date	Blood product	Clinical details/symptoms after transfusion
1	01 November 2019	PRBCs	Patient on day 10 of chemotherapy cycle 3, receiving gentamicin. Vitally stable the following day.
2	02 November 2019	Platelets	Patient on day 11 of chemotherapy cycle 3, on gentamicin. Vitally stable for >48 h. On 03 November 2019, spiked fever of 38.2 °C at 8 p.m.; right ear bleeding noted by ENT; diarrhea observed.
3	05 November 2019	PRBCs	Patient on day 14 of chemotherapy cycle 3, on gentamicin (day 6). Vitally stable; no ear bleeding or diarrhea. Discharged on 12 November 2019 in good health.
4	11 December 2019	PRBCs	Vitally stable, afebrile for >5 days.
5	01 January 2020	Platelets	Vitally stable, afebrile for >72 h.
6	05 January 2020	PRBCs	Vitally stable, afebrile for >24 h. Discharged.
7	28 January 2020	PRBCs/Platelets	Patient on day 8 of chemotherapy cycle 6. Hemodynamically stable until days 10–12; developed persistent febrile episodes and tachypnea.
8	01 February 2020	Platelets	Patient on day 13 of chemotherapy cycle 6. Spiking temperatures noted; on 02 February 2020, first febrile tonic seizure, aborted with lorazepam. Continued antibiotics. On 03 February 2020, the temperature improved (37.7 °C at 6 a.m.). On 04 February 2020, a second seizure occurred during Keppra infusion, with a temperature spike of 38.7 °C, tachypnea, and tachycardia of 142 bpm. Dengue serology sent on 06 February 2020; WNV serology sent on 10 February 2020.
9	13 February 2020	Platelets	Patient on day 25 of chemotherapy cycle 6, afebrile for 7 days. On 17 February 2020, 4 days post-transfusion, the temperature spiked to 38 °C. Patient on ceftriaxone remained afebrile for 72 h afterward.
10	22 February 2020	PRBCs	Transfusion requested by the consultant, but not recorded in the patient plan by the assistant consultant on the same day.

## Discussion

Numerous reports have documented WNV transmission through blood and organ transplantation, which can result in neuroinvasive infection and death in many recipients (6, 9, 14, 15). The present case of Ewing’s sarcoma undergoing treatment highlights the clinical and transfusion-related challenges posed by WNV infection in immunocompromised individuals. It underscores the potential risk of WNV transmission through donated blood in endemic or emerging regions such as Saudi Arabia. Although West Nile fever is asymptomatic in most cases, less than 1% of patients develop encephalitis, myelitis, or meningitis, which can be fatal. In immunosuppressed patients, the clinical presentation and radiographic manifestations differ from those observed in non-immunosuppressed patients. In immunosuppressed individuals, including patients receiving chemotherapy, antibody responses to WNV may be delayed or attenuated, resulting in reduced sensitivity of serological testing during early infection. Consequently, these patients are more likely to have PCR-detectable WNV nucleic acid for a prolonged period compared with immunocompetent individuals, highlighting the diagnostic importance of nucleic acid testing in this population. In immunosuppressed patients, prompt identification of the causative pathogen and initiation of targeted therapy is essential to prevent potentially lethal outcomes. The clinical course aligns with documented neuroinvasive manifestations of WNV. While approximately 75% of infections remain asymptomatic, less than 1% of cases progress to severe neurological involvement, such as encephalitis, meningitis, or acute flaccid paralysis, primarily in elderly or immunocompromised individuals (16–19). The child’s recurrent seizures, MRI findings of thalamic and midbrain signal changes, and laboratory confirmation of WNV infection are compatible with WNV encephalitis and are similar to findings in earlier reports of transfusion-associated cases in immunosuppressed patients (9).

A patient with Ewing’s sarcoma receiving chemotherapy may develop specific neurological complications, and infection with WNV may then lead to persistence of symptoms, such as tremors and bradykinesia, for years (20), potentially leading to confusion for the treating medical oncologist, who may attribute the symptoms to chemotherapeutic neurotoxicity. WNV infection has no clinical treatment, and no approved vaccines are available. Because WNV can be transmitted through the transfusion of infected blood products, blood safety policies recommend temporary deferral of blood donation following confirmed or suspected infection to reduce the risk of transfusion-associated transmission. These precautions are particularly important because asymptomatic or presymptomatic donors may harbor detectable viral nucleic acid despite the absence of clinical illness, reinforcing the importance of appropriate donor screening strategies based on local epidemiology (21).

The detection of anti-WNV antibodies in 13.3% (4/30) of randomly screened blood donors in this study strongly supports transfusion as a probable epidemiological source; however, a definitive causal relationship cannot be established. A study by Pealer et al. in the United States found that 43% of blood recipients who acquired WNV were immunocompromised (22). Screening of blood for WNV antibodies is now routinely implemented in both the United States and Europe in connection with frequent seropositivity in these regions (23). This finding is consistent with international reports where WNV transmission through blood components has been well documented, particularly during seasons of peak mosquito activity (21). Several studies from North America and Europe have reported asymptomatic WNV viremia among blood donors, with the virus capable of surviving standard blood storage conditions and infecting recipients, especially those who are immunocompromised (24).

In Saudi Arabia, serological evidence of WNV exposure has been documented among both humans and animals, suggesting silent local circulation of the virus (25). The confirmed case reported in Jeddah in 2020 further establishes the region as an area of emerging concern (26). Given the tropical climate, the presence of *Culex* mosquitoes, and increasing international travel and migration, ecological conditions favor continued WNV transmission and expansion of its vector range (27).

The present case can be discussed in two ways: (a) the immediate need for integration of WNV screening into routine pretransfusion testing protocols, particularly for high-risk groups such as oncology, transplant, and immunocompromised patients, and (b) the importance of enhancing national surveillance systems to monitor arboviral infections beyond dengue and chikungunya. Although IgM antibody capture ELISA is widely used for the clinical diagnosis of WNV infection, antibody testing alone is insufficient for blood donor screening due to the window period during which donors may be viraemic but seronegative. Therefore, nucleic acid testing (NAT) for WNV RNA is considered the preferred method for transfusion safety, as implemented in the United States and parts of Europe during transmission seasons. In developing countries, implementation in resource-limited settings remains sporadic due to cost and infrastructure barriers (28). Nonetheless, targeted seasonal screening or selective testing based on donor travel history and epidemiological risk could substantially reduce transfusion-associated WNV transmission.

The case also underscores the need for greater clinical awareness among physicians. WNV infection should be considered in the differential diagnosis of febrile illness with neurological manifestations in transfused patients, especially when dengue, malaria, and bacterial causes are excluded. Early recognition allows supportive management and appropriate infection control measures, as no specific antiviral therapy or licensed vaccine is currently available for WNV (29).

From a public health perspective, the detection of anti-WNV antibodies in healthy donors signifies subclinical viral circulation within the community. Strengthening vector control programs, improving donor eligibility screening, and enhancing diagnostic capacity for arboviruses are essential preventive steps. Collaboration between transfusion centers, infectious disease units, and public health authorities will be crucial to prevent similar cases in the future. Implementation of NAT screening has previously reduced transfusion-transmitted WNV risk in several countries, highlighting the importance of molecular screening during periods of increased viral circulation.

## Limitations

Detailed serological titers, PCR assay parameters, transfusion records, donor traceback findings, and specific serological or nucleic acid testing results were not available due to limitations related to retrospective data access. Consequently, transfusion-associated transmission could not be definitively established, and this represents an important limitation of the present report. Confirmatory plaque reduction neutralization testing (PRNT) was not performed to differentiate WNV-specific antibodies from potential cross-reactive flavivirus antibodies, representing an additional diagnostic limitation.

## Conclusion

This case was potentially associated with transfusion exposure and contributes to the growing body of evidence highlighting the need for careful consideration of transfusion-transmitted WNV infection, particularly in immunocompromised patients, while acknowledging that a definitive source of infection could not be established. Routine donor screening, clinician vigilance, and enhanced surveillance are imperative to safeguard vulnerable populations from this emerging infection.

## Recommendations

1. Targeted screening of blood products for high-risk recipients should be carried out, particularly in immunosuppressed patients, oncology cases, and transplant recipients.
2. Donor risk assessment should be enhanced by including screening for recent febrile illness, travel history to endemic areas, and mosquito exposure.
3. Periodic seroepidemiologic surveys among blood donors should be conducted to monitor silent circulation of WNV.
4. Adoption of a phased or sentinel-based screening model may provide a cost-effective and evidence-based approach in Saudi Arabia.
5. Nucleic acid testing (NAT) for WNV RNA should be considered the preferred strategy for donor screening, as it allows the detection of active viremia prior to seroconversion.

## Data Availability

The original contributions presented in the study are included in the article/Supplementary material, further inquiries can be directed to the corresponding authors.
